# Biomimetic “Cactus Spine” with Hierarchical Groove Structure for Efficient Fog Collection

**DOI:** 10.1002/advs.201500047

**Published:** 2015-05-26

**Authors:** Fan Bai, Juntao Wu, Guangming Gong, Lin Guo

**Affiliations:** ^1^ Key Laboratory of Bio‐Inspired Smart Interfacial Science and Technology of Ministry of EducationSchool of Chemistry and EnvironmentBeihang UniversityBeijing100191P. R. China

**Keywords:** biomimetic materials, cactus spines, electrospinning, fog collection, nanogrooves

## Abstract

**A biomimetic “cactus spine” with hierarchical groove** **structure** is designed and fabricated using simple electrospinning. This novel artificial cactus spine possesses excellent fog collection and water transportation ability. A model cactus equipped with artificial spines also shows a great water storage capacity. The results can be helpful in the development of water collectors and may make a contribution to the world water crisis.

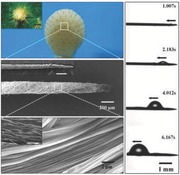

This is an open access article under the terms of the Creative Commons Attribution License, which permits use, distribution and reproduction in any medium, provided the original work is properly cited.

The water crisis is an urgent problem around the world. How to utilize the potential water has attracted intensive attention. In nature, several creatures have the ability to collect water from humid fog, which is an efficient method to turn the potential water into availability. For instance, the beetles in the desert can take advantage of the hydrophilic and hydrophobic structures of its back to collect water.^1^, ^2^, ^3^ Also, the spider silk is able to harvest abundant water by using a periodic joints and spindle‐knots structure.^4^, ^5^ Moreover, the cactus, a kind of well‐known drought‐resistant plant, also shows an intriguing capability of fog collection and water transportation. Research about the cactus has revealed that its water‐collection ability derives from the hierarchical groove structure on the surface of the cactus spine, that can endow itself with a Laplace pressure gradient and wettability gradient.^6^ And the cooperation of these two gradients can provide a driving force to transport the water droplet from the tip to the base,^7^, ^8^, ^9^ where it can be absorbed by trichomes rapidly. Inspired by this directional water transportation property, several fog collectors or water/oil separators have been developed.^10^, ^11^, ^12^, ^13^

However, it was found that most researches only focused on how to obtain a surface with wettability gradient, but ignored the similarity of micro/nanostructure, namely, hierarchical groove structure. For the cactus, evolution of this distinctive structure should be the best choice of nature. Thus, we speculated that this groove structure might play a more important role in the process of water transportation. Unlike the inconvenient preparation methods in previous reports, electrospinning (ES), a facile technique to build and control the hierarchical micro/nanostructure,^14^, ^15^, ^16^, ^17^, ^18^ can be utilized to mimick the groove structure. Among the ES materials, polyimide (PI) is a kind of high‐performance polymer with remarkable thermal stability and mechanical strength, so that it could be electrospun into fibers with different surface structure.^19^, ^20^, ^21^ In this work, by means of ES and a sacrificial template method, we have designed and fabricated an artificial “cactus spine” with hierarchical groove structure. Owing to these special structures, the artificial spine showed an excellent fog collection and water transportation performance, confirming the vital function of the groove structure of a real cactus spine. This work may pave a new avenue to develop water collectors and have a potential application in solving global water crisis.

To obtain the special nanogroove structure on the surface of electrospun PI fibers, we chose an easily decomposition polymer, polystyrene (PS), to serve as sacrificial template. Detailed experimental procedures were provided in the Supporting Information. The mixed solution of PS and poly(amic acid) (PAA, the precursor of PI, as shown in Scheme S1, Supporting Information) was vigorously stirred to get a uniform mixture and then electrospun into composite fibers at room temperature. In the ES jets, molecule chain aggregations of PS were stretched constantly and distributed uniformly along the longitudinal direction of fibers (as shown in **Scheme** **1**a). Then, during the following imidization process, sacrificial PS was decomposed and resulted in numerous nanogrooves on the surface of PI fibers (as shown in Figure S1, Supporting Information).

**Scheme 1:**
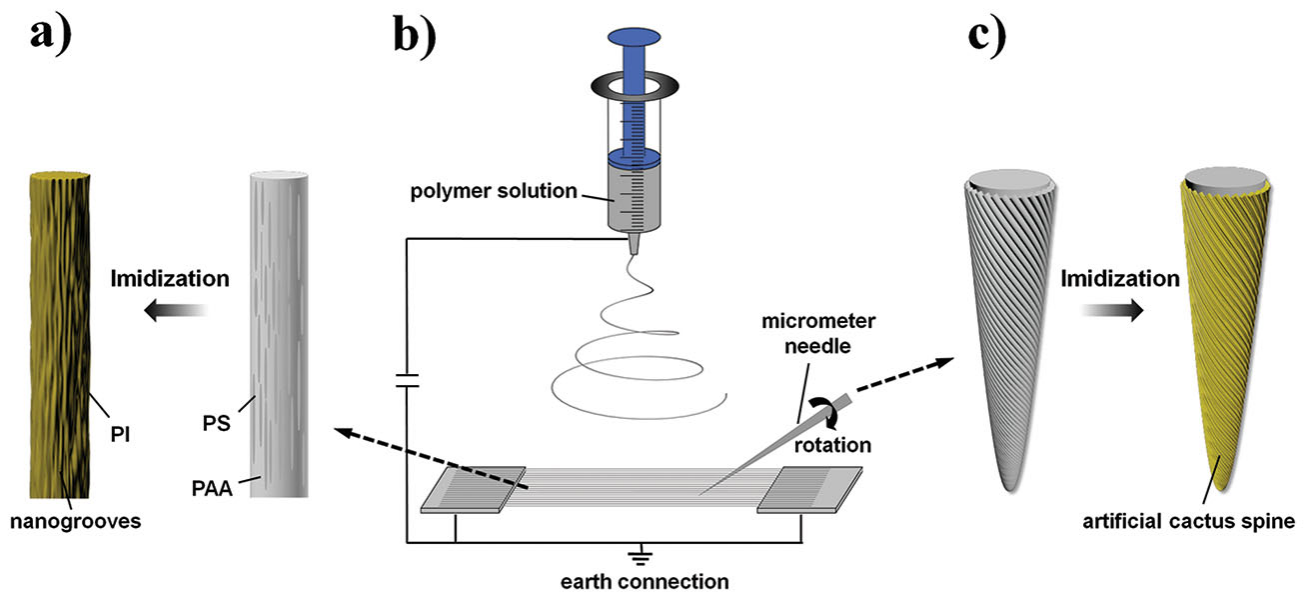
Fabrication procedure of the artificial cactus spine by combining ES with sacrificial template method. a) After a thermal imidization treatment, the electrospun PAA‐PS composite fiber could be turned into nanogrooved PI fiber. b) Under the action of the electric field force, the as‐prepared composite fibers would parallel cross the electrode gap. Then, one silver needle was rotated along the aligned fibers at a fixed angle to cover fibers on the surface. c) In a following imidization process, PS was removed, and an artificial spine with hierarchical groove structure was obtained.

In order to mimick the surface structure of a real cactus spine, the PAA‐PS composite fibers were electrospun to span across the electrode gap. Then, a micrometer silver needle was rotated along the aligned fibers at a fixed angle to cover fibers on its surface. After an imidization treatment, an artificial cactus spine with hierarchical groove structure could be obtained (as shown in Scheme 1b,c). The morphology of the prepared spine was shown in **Figure** **1**. It could be observed that the artificial spine possessed a conical shape, and the nanogrooved fibers covered over the silver needle along a spiral direction. Figure 1c,d showed that the artificial spine had a sparse fiber bundle near the base and a tight fiber bundle near to tip, which were similar to the first‐level grooves of a real cactus spine.^6^ Also, the nanogrooved structures on the surface of PI fibers also presented a analogous morphology to the second‐level grooves of a real cactus spine (as shown in the inset image of Figure 1). Thus, we managed to mimick the hierarchical groove structure of cactus spine from microscopic to macroscopic.

**Figure 1:**
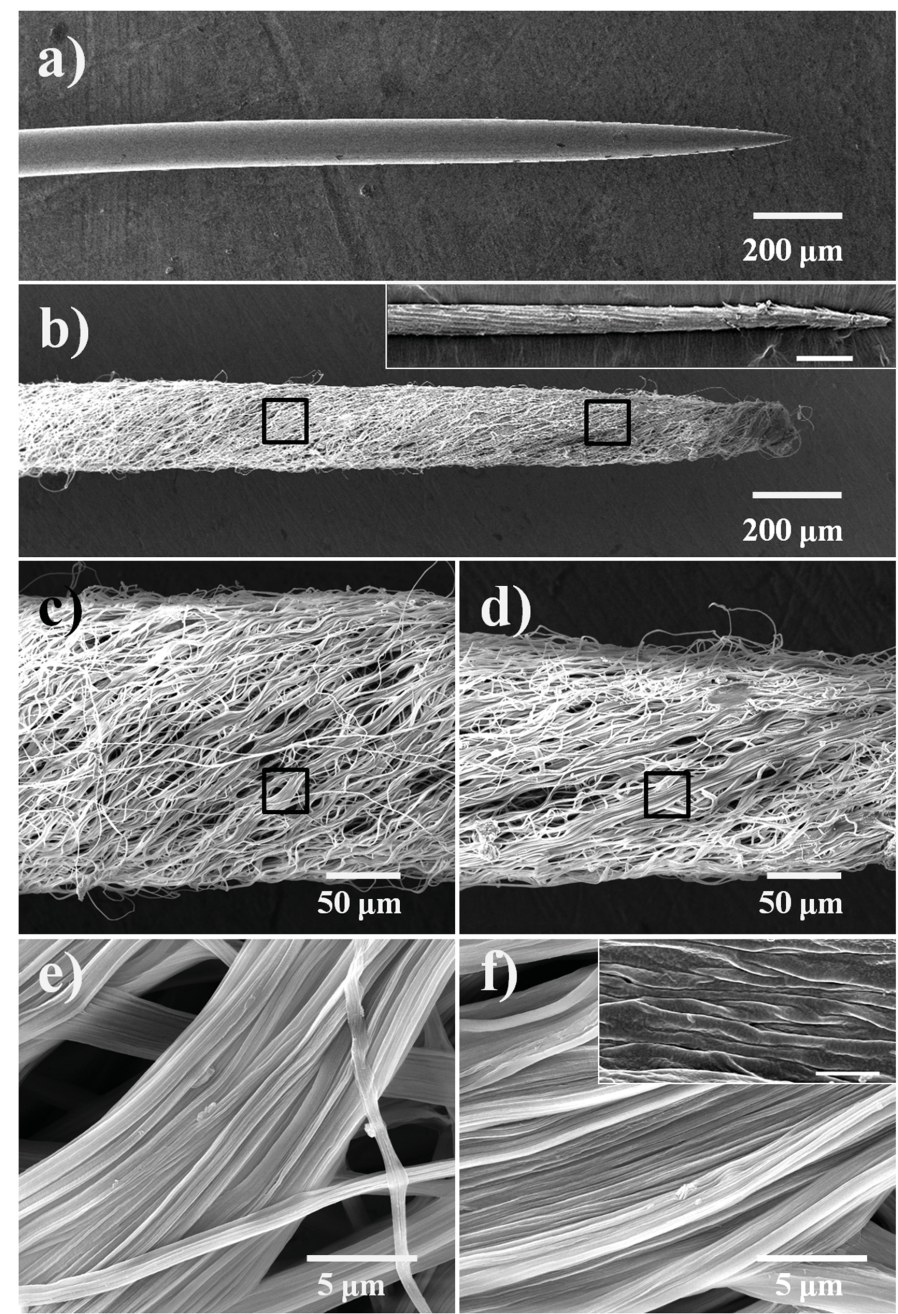
Morphologies of the artificial cactus spine with hierarchical groove structure. SEM image of a) bare silver needle and b) the artificial spine. Inset: SEM image of a real cactus spine. Scale bar: 100 μm.^6^ c) Enlarged SEM image of the left outlined areas of panel (b), the sparse fiber bundles. d) Enlarged SEM image of the right outlined areas of panel (b), the tight fiber bundles. e,f) Enlarged SEM image of the outlined area of panels (c,d), the nanogrooved PI fibers. Inset: SEM image of the second‐level groove structure of a real cactus spine. Scale bar: 2 μm.^6^

A fog collection and water transportation test was taken to measure the effect of nanogrooved structures on the artificial spine by using a saturated fog flow with a velocity of about 55–60 cm s^–1^. The artificial spine was placed at different tilt angles (90°, 45°, 0°, –45°, and –90°) to prove its effectiveness of water‐transportation ability under multiple directions. From **Figure** **2**, we could observe that the tiny water droplets arose at the tip of spine and merged with each other soon, forming a bigger one. For each case, even when the tip of spine pointed down, the growing water droplet was directionally driven from the tip to the base. This fog collection and water transportation behavior was just similar to that of a real cactus spine. In the present work, the movement speed of water droplet could come to about 360 μm s^–1^. So we inferred that the nanogroove structure might have a great influence on the behavior of water transportation. Then, as a comparison, other two artificial “spines,” with bare silver needle and smooth PI fibers, were prepared and measured under the same conditions (as shown in Figure S2, Supporting Information). The spine with smooth PI fibers also presented a fog collection and water transportation performance, but the movement rate of water droplet was only about 159 μm s^–1^, that was far smaller than the artificial spine with hierarchical groove structure. Besides, for the bare silver needle, the water droplets could only arise and merged with each other at the tip part, without being transported directionally (as shown in Figure S3, Supporting Information). The above results clearly illustrated that the introduction of nanogroove structure could improve the water transportation ability of the artificial cactus spine significantly.

**Figure 2:**
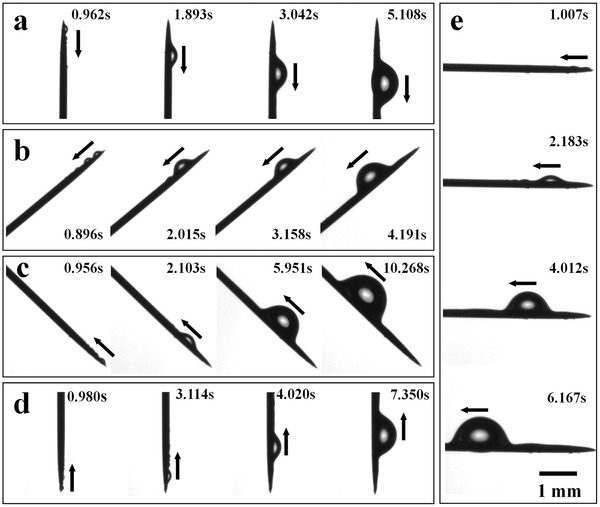
Optical microscopic observation of the directional water movement on the artificial cactus spine at different angles. a–e) An artificial spine was placed in a fog flow at the following angles: 90°, 45°, 0°, °45°, and –90°, respectively. In a very short time, the water droplet could be collected and driven from the tip to the base of the spine.

The remarkable water transportation behavior can be explained as follows: As the diameter decreased, the fibers would capture the moisture easier. When a fog flow passed by, water droplets arose and merged with each other on the surface of nanogrooved PI fibers. Then, the water preferred to be trapped by internal fiber bundle, which could provide a capillary force to the water droplets (as shown in Scheme S2, Supporting Information). On the other hand, the water droplet that was deposited on the surface of the spine could be driven by a Laplace pressure. Also, the micro/nanometer groove structure of artificial spine modified the air–liquid–solid three‐phase contact line (TCL) of the water droplet (as shown in **Scheme** **2**a–c). For the water droplet on the surface of a bare silver needle, the Laplace pressures difference between the two opposite sides had a relationship as

(1) $$\Delta P\sim \gamma_{\mathrm{water}}\left(\frac{1}{R_{1}^{}}-\frac{1}{R_{2}^{}}\right)$$

where *R*_1_ and *R*_2_ are the radii of the TCLs at the two opposite sides of conical spine, and *γ*_water_ is the surface tension of water. Then as shown in Scheme 2d,e, for the water droplet on artificial spine, the TCLs could be divided into several parts by the nanogrooved PI fiber, and the Laplace pressure difference at each part of the two opposite sides could be represented as

(2) $$\Delta P\prime \sim \gamma_{\mathrm{water}}\left(\frac{1}{R_{1}\prime }-\frac{1}{R_{2}\prime }\right)$$

where *R_1_'* and *R_2_'* are the radii of the divided parts of TCLs at the two opposite sides. Because this division could raise the local radius of the TCLs and the local curvatures, the Laplace pressures difference was enlarged by the nanogrooved PI fibers. Moreover, it was worth to be mentioned that the numerous nanogrooved structures on the surface of fibers could further divide the TCLs significantly, supplying an additional driving force to the water droplet, or equivalently, these nanogrooves could generate smaller initial radii for the driven water droplets. Therefore, the cooperation of internal capillarity action and superficial Laplace pressure endowed the artificial cactus spine with an excellent water transportation property.

**Scheme 2:**
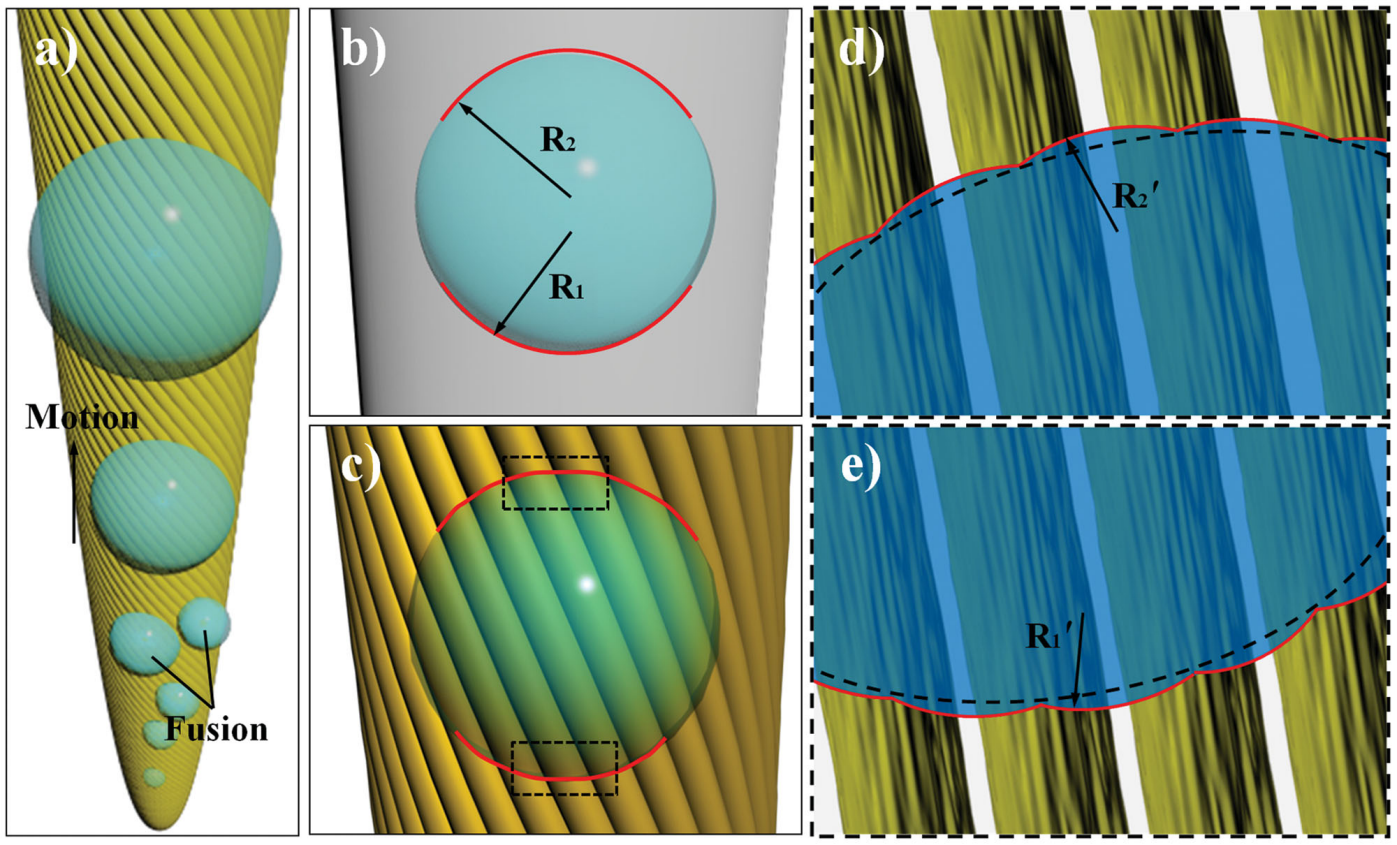
Directional movement mechanism of the collected water droplet on the artificial cactus spine. a) Schematic of the water collection process. The deposited water droplets grow and are driven from the tip to the base. b,c) Mechanism of water transfer on the surface of bare silver needle and artificial spine. d,e) Enlarged schematic of panel (c). The TCLs are divided into several parts by the PI fibers with nanogrooves.

A row of artificial spines were fixed in a piece of water‐saturated sponge to preliminary test the fog collection ability (as shown in **Figure** **3**a,b). Under a fog flow, the water droplet was deposited at the tip and driven to the base, so that it would be absorbed by the sponge constantly. Once the sponge was overloaded with water, the excess water would flow down along the bevel. Furthermore, a cactus‐like model that was consisted of 180 artificial spines and a spherical sponge was prepared to further illustrate the fog collection ability visually (as shown in Figure 3c). In 15 min, the model cactus could harvest about 1.3 mL of water (as shown in Figure 3e,f). Then we could estimate that the average water collection rate of a single artificial spine was approximately 0.3 μL min^–1^. This results indicated that we could meet the basic water requirement for an adult by using 200 model cactus to collect water from fog flow in about 2.4 h.^22^ Consequently, in line with the real cactus, the introduction of nanogroove structure could be used to improve the fog collection and water transportation ability, and the man‐made model cactus with hierarchical grooved spines also showed a high water‐storage capacity.

**Figure 3:**
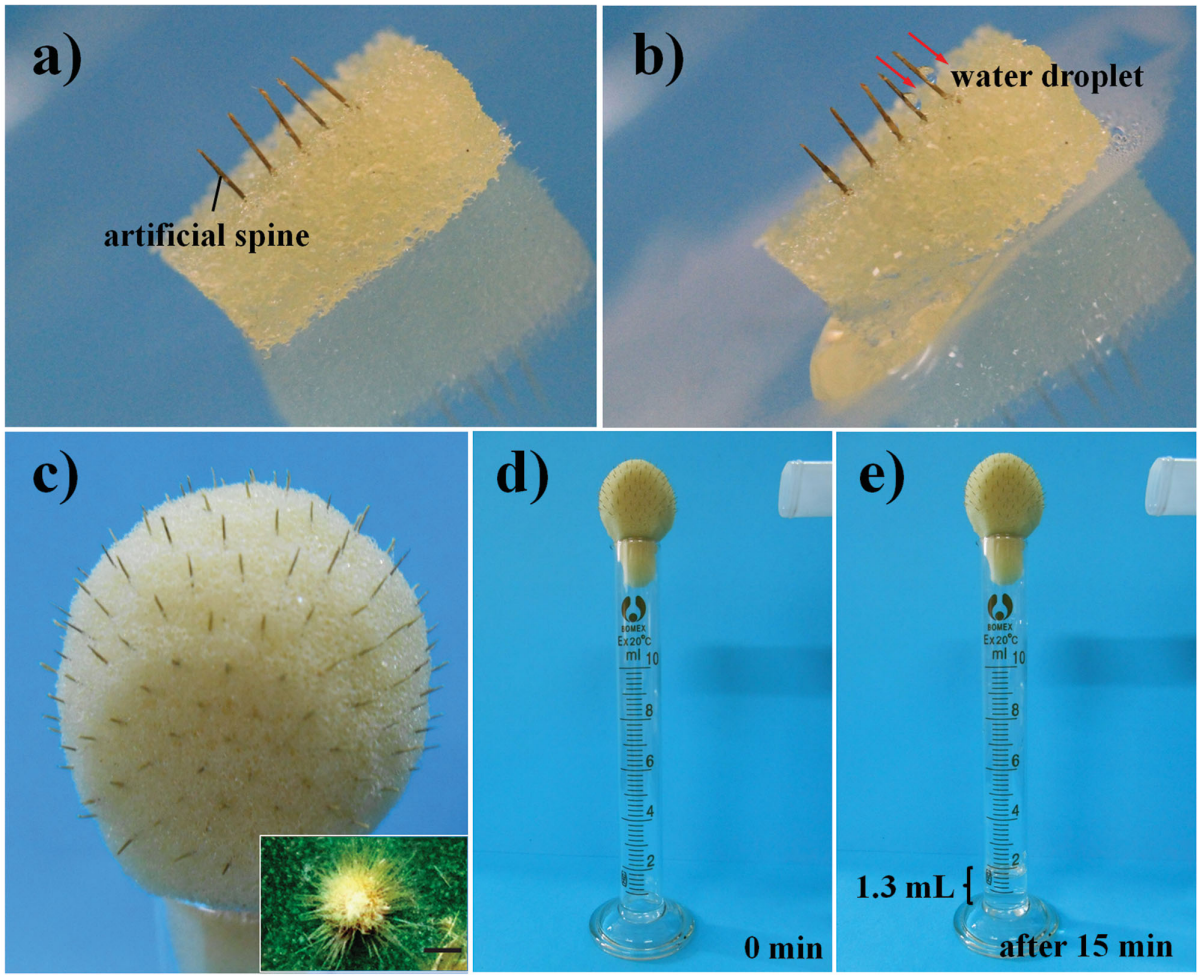
a,b) Fog collection test of an artificial spine array. The spine array was fixed on a piece of water saturated sponge, and it could collect water under a fog flow with a velocity of about 55–60 cm s^−1^. c) Photograph of the model cactus equipped with 180 artificial spines. Inset: Optical image of a cluster of spines in a real cactus.^6^ Scale bar: 100 μm. d,e) Fog collection ability test of the prepared model cactus. The model cactus was fixed on a cylinder, and the velocity of fog flow was around 55–60 cm s^−1^. The distance between the model cactus and fogging jet was about 4 cm.

In summary, we have designed and fabricated a biomimetic “cactus spine” with hierarchical groove structure and excellent water transportation performance. The deposited water droplet can be transported at an amazing velocity of about 360 μm s^–1^, and the man‐made model cactus equipped with artificial spines also presents great water‐storage property. In accord with a real cactus, it can be concluded that the hierarchical grooved structures make a great contribution to fog collection and water transportation property. So our work is helpful to develop novel water collectors, and it may have a potential application in solving world water crisis.

## Supporting information

As a service to our authors and readers, this journal provides supporting information supplied by the authors. Such materials are peer reviewed and may be re‐organized for online delivery, but are not copy‐edited or typeset. Technical support issues arising from supporting information (other than missing files) should be addressed to the authors.

Supplementary
