# Supplementary material for: Biomimetic “Cactus Spine” with Hierarchical Groove Structure for Efficient Fog Collection
Source: Adv Sci (Weinh). 2015 May 26;2(7):1500047. doi: 10.1002/advs.201500047 (PMC5115433; doi:10.1002/advs.201500047)
Supplement: Supplementary file 1 — Supplementary [file ADVS-2-0i-s001.pdf]

Copyright WILEY-VCH Verlag GmbH & Co. KGaA, 69469 Weinheim, Germany, 2015.

ADVANCED SCIENCE
Open Access

Supporting Information

for *Adv. Sci.*, DOI: 10.1002/advs. 201500047

Biomimetic "Cactus Spine" with Hierarchical Groove Structure for Efficient Fog Collection

Fan Bai, Juntao Wu, Guangming Gong, and Lin Guo**

Supporting Information:

Biomimetic "Cactus Spine" with Hierarchical Groove Structure for Efficient Fog Collection

Fan Bai, Juntao Wu*, Guangming Gong, and Lin Guo*

[*] F. Bai, Dr J. Wu, G. Gong, Prof. Dr. L. Guo
Key Laboratory of Bio-Inspired Smart Interfacial Science and Technology of Ministry of Education
School of Chemistry and Environment, Beihang University
Beijing, 100191, PR China
E-mail: wjt@buaa.edu.cn; guolin@buaa.edu.cn

Experimental Section

Materials

4,4'-oxydianiline (ODA; purified by sublimation), pyromellitic dianhydride (PMDA; recrystallized before use), and silver needle were purchased from LanYi Company in Beijing. Polystyrene (PS; average Mw ~192000) was purchased from Sigma-Aldrich and N,N-Dimethylformamide (DMF, ≥99.5%, dehydrated by molecule sieves) were purchased from Beijing Chemical Co.

Preparation of a artificial cactus spine and model cactus

First, ODA, PMDA and DMF were used to prepare a 10 wt% PAA solution by a typical polymerization[1], and a 20 wt% PS solution was prepared by dissolving PS in DMF directly. The two solutions were mixed in a mass ratio of 1:1 and strongly stirred at room temperature for at least 5 h to yield a homogeneous mixed solution. Second, the mixed polymeric solution was electrospun to span across two grounding electrodes under a 15 kV voltage (Spellman SL50P60, USA). The distance between spray nozzle and collecting electrodes was around 15 cm. Third, a micrometer silver needle was rotated along the aligned composite fibers at a fixed angle, so that the fibers could cover on surface of needle. And the as-prepared artificial spine was heated at 120, 150, 180, 250, 300, and 350 ^{o}C to undergo an imidization process,

which could turn PAA into PI. Furthermore, 180 prepared artificial spines were assembled on a spherical sponge to prepare a man-made model cactus.

Characterization

The morphology test was characterized by an environmental scanning electron microscope (ESEM, Quanta 250 FEG). An ultrasonic humidifier (Goai, G0-2028, China) was used to generate the fog flow with a velocity of around 55-60 cm/s at room temperature, which was vertically fixed to the artificial spine. The distances between the jet and artificial spine/model cactus were both about 4cm. The sponge was waterlogged so that the collected water droplets could be absorbed and flow down easily, and it just could collect about 0.5 mL water in 15 min. The whole fog collection and water transportation process was observed and recorded by an OCA20 machine with 25 frames per second.

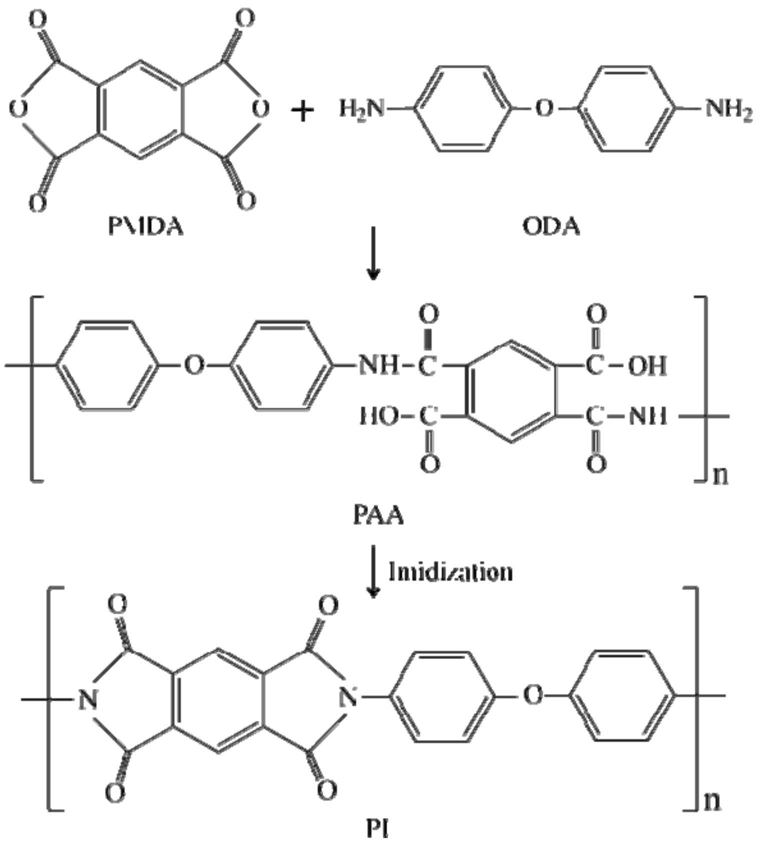

Scheme S1. Process for synthesizing the PI. The raw materials, PMDA and ODA, were generated into PAA by a condensation reaction. And after a thermal imidization treatment, the PAA can be turned into PI.

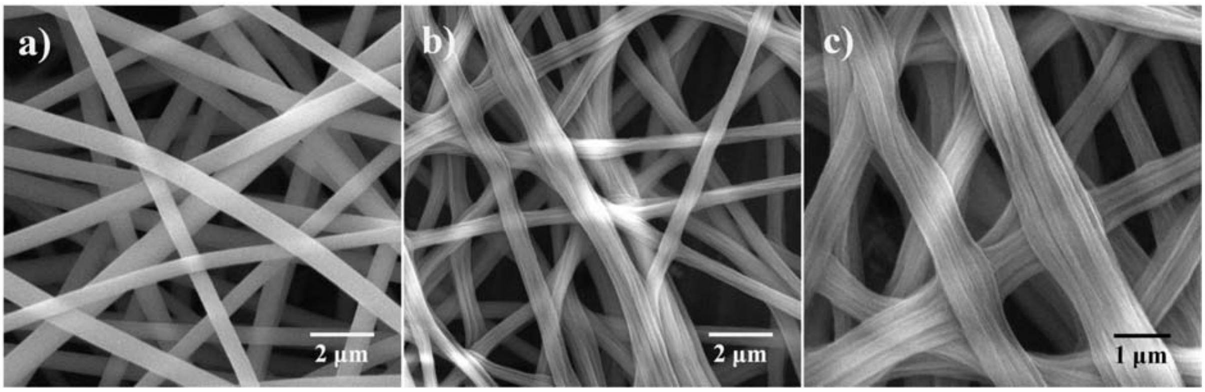

Figure S1. SEM images of the electrospun (a) PAA-PS composite fibers with smooth surface and (b) PI fibers with nanogroove structure after a high temperature treatment. (c) High magnification of part b.

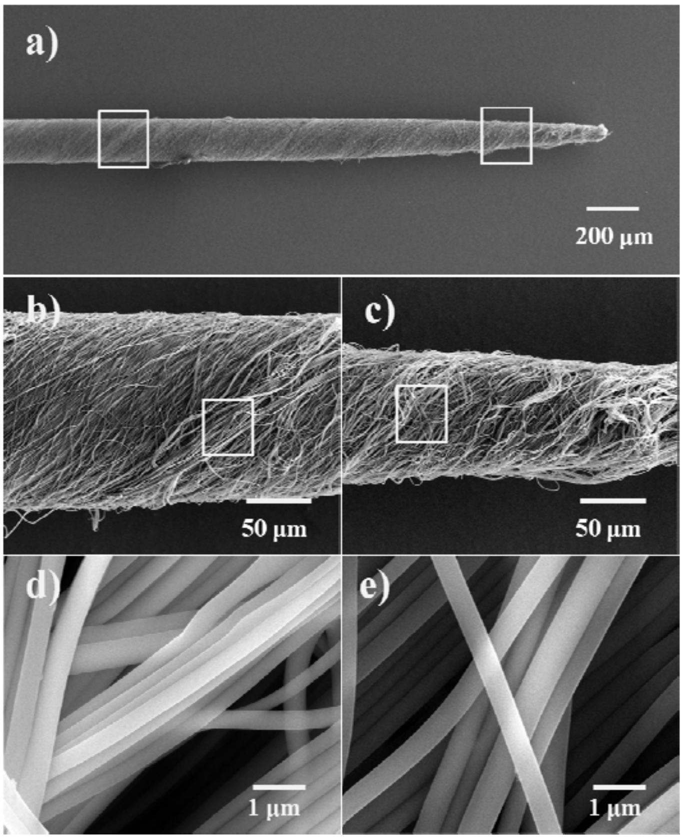

Figure S2. Morphology of the prepared "spine" with smooth PI fibers. a) SEM image of the spine. b-c) Enlarged SEM image of the outlined area of part a. d-e) Enlarged SEM image of the outlined area of part b and part c, the PI fibers with smooth surface.

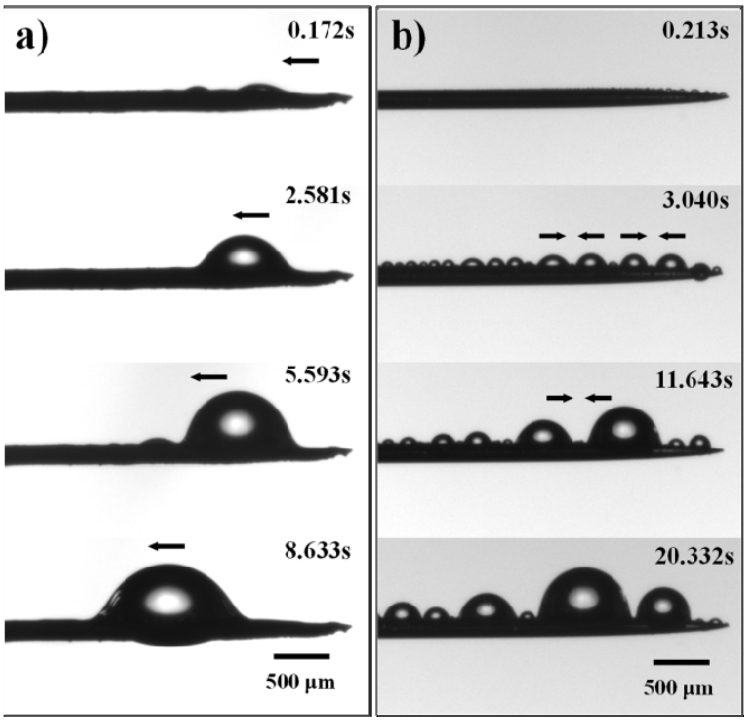

Figure S3. a) For prepared "spine" with smooth PI fibers, water droplet can be collected at the tip and transported from tip to base slowly. b) While water droplets on surface of a bare silver needle only merge with each other, but not transported directionally.

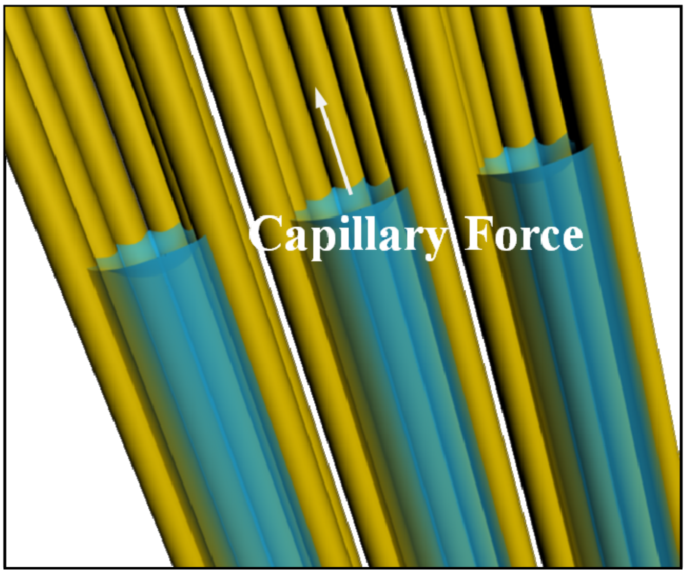

Scheme S2. Water inside the fiber bundle can be driven by a capillary force.

Reference

[1] J. Wu, S. Yang, S. Gao, A. Hu, J. Liu, L. Fan, *Eur. Polym. J.*, 2005, **41**, 73-81.
